# Reporting of psychotherapeutic methods in psychedelic treatments: on the road to ethical, professional and regulatory oversight

**DOI:** 10.1192/j.eurpsy.2025.64

**Published:** 2025-08-26

**Authors:** A. Oliveira-Maia

**Affiliations:** 1 Champalimaud Foundation; 2 NOVA Medical School, Lisbon, Portugal

## Abstract

**Abstract:**

The potential of psychedelic substances to treat mental illness is of significant clinical and societal interest, leading to academic and industry-based research to test their effects. Partly, such research was conducted to fulfil requirements of government agencies such as the European Medicines Agency (EMA) and the Food and Drug Administration (FDA), that have started defining requirements and pathways to regulate psychedelic treatments. It is expected that such requirements will involve elements related to psychotherapeutic components of such treatments, which will require standardized reporting of such methods. Here, I will present the results of a systematic review summarising the quality of reporting on psychological interventions in original studies on psychedelic-assisted psychotherapy. We reviewed 45 studies assessing psilocybin, 3,4-methylenedioxymethamphetamine (MDMA), lysergic acid diethylamide (known as LSD), or ayahuasca, for the treatment of mental disorders. Our findings support that psychological interventions were done heterogeneously across studies, and completeness of information reported about these interventions was mostly low, according to an adaptation of the Template for Intervention Description and Replication checklist. In studies including MDMA, psychotherapy was more homogeneous and more procedural details were provided. We thus propose that improved reporting on psychological interventions of psychedelic treatments are necessary to support replicability, generalisability, and accurate interpretation of research. Furthermore, improved reporting practices are expected to enhance feasibility and safety of future clinical research and real-world implementation of treatments.

**Disclosure of Interest:**

None Declared

